# Exploration and Analysis of GaN-Based FETs with Varied Doping Concentration in Nano Regime for Biosensing Application

**DOI:** 10.3390/bios15090613

**Published:** 2025-09-16

**Authors:** Abhishek Saha, Sneha Singh, Rudra Sankar Dhar, Kajjwal Ghosh, A. Y. Seteikin, Amit Banerjee, I. G. Samusev

**Affiliations:** 1 Department of ECE, National Institute of Technology, Aizwal 796012, Mizoram, India; abhishek.ece.phd@nitmz.ac.in (A.S.); dt23ec001@nitmz.ac.in (S.S.); 2 Department of ECE, Dream Institute of Technology, Kolkata 700104, West Bengal, India; abhishek_saha@ditkol.com; 3 Research and Education Center for Fundamental and Applied Photonics & Nanophotonics, Immanuel Kant Baltic Federal University, 236000 Kaliningrad, Russia; seteikin@mail.ru; 4 Microsystem Design-Integration, Laboratory of Physics Department, Bidhan Chandra College, Asansol 713303, West Bengal, India

**Keywords:** nanobiosensor, GaN, bio detector, sensitivity, linearity

## Abstract

This study conducts a comprehensive examination of a GaN channel-based nanobiosensor featuring a dielectrically modulated trigate FinFET structure, incorporating both uniform and Gaussian channel doping. The proposed device incorporates a nanocavity structure situated beneath the gate region, intended for the analysis of diverse biomolecules in biosensing applications. The proposed biosensor employs HfO_2_ as the gate dielectric, characterized by a dielectric constant of 25, leading to an enhanced switching ratio for the device. This study examines the electrical properties relevant to biomolecule identification, including the switching ratio, DIBL, threshold swing, threshold voltage, and transconductance. The sensitivity of these properties concerning the drain current is subsequently assessed. Enhanced sensitivity increases the likelihood of detecting biomolecules. The electrical property of a biomolecule is examined in the absence of another biomolecule within the cavity. The apparatus is designed to detect neutral biomolecules. Simultaneously, further investigational research has been undertaken regarding the linearity behavior of GAA FET, nanobiosensors, and dielectrically modulated TGFinFET. This study’s results have been compared with those of GaN-based FinFET and GaN SOI FinFET technologies. The data indicates approximately ∼103% and ∼42% improvements in *I*_*OFF*_ and Switching ratio, respectively, when compared to IRDS 2025. The nanobiosensor (GAA FET) demonstrates enhanced linear performance concerning higher-order voltage and current intercept points, including VIP2, VIP3, IIP3, and P1dB.

## 1. Introduction

The current semiconductor landscape is characterised by a continuous trend of modernizing device structures via material modifications, resulting in ongoing transistor scaling. Short channel effects (SCEs) result from the downscaling of devices [1] and optimizing SCEs beyond the 20 nm technology node presents a significant challenge. The SCEs impose a considerable effect on electrical characteristics at the nanoscale, including mobility degradation, alterations in threshold voltage, and an increase in off-current leakages [2] which results in the generation of hot carriers in the nano regime. Additionally, these hot carriers generate trap charges at the Si-SiO_2_ interface of the MOSFET structure, which considerably enhances short-channel effects (SCEs) and lead to subsequent degradations in device performance. Numerous solutions are currently under investigation to enhance device performance and minimize variability [3]. These include the development of nanoscale gate-all-around field-effect transistors (GAA FETs), the implementation of high-k gate materials [4], the optimization of channel doping profiles [5], and the utilization of multi-gate structured field-effect transistors (FETs) [6]. Keeping this in mind, a significant focus of research has been directed towards the detection of biomolecules these days. The identification of biomolecules plays a crucial role in the early diagnosis of diseases, including Alzheimer’s disease, ovarian cancer, and specific viral infections. Conventional methods require a significant duration for the detection of DNA, RNA, glucose, biotin, streptavidin, and blood [7]. This is due to the fact that they are all biological substances. Researchers are currently engaged in the development of new FET sensors aimed at reducing the time necessary for detection. Conventional FETs are miniaturized to the nanoscale, resulting in a reduction of the SiO_2_ layer utilized as the gate dielectric. This reduction leads to an increase in gate leakage current and power dissipation [8,9]. In order to tackle these effects, high-k dielectric serves as an alternative material within the current Fin FET technology. This work employs GaN material as the channel material instead of Silicon (Si), while HfO_2_ serves as the gate dielectric in place of SiO_2_. The gate in a FinFET encompasses the channel region on three sides, while in Nanostructure, the gate surrounds the channel, thereby categorizing the device as a GAA device. This architecture results in enhanced electrostatic control of the transistor [10], increased transistor switching speeds, and adequate driving currents within a reduced footprint. Consequently, the GAA FET is positioned as a promising new technology [11], featuring gates on all sides. GAA FET technology is anticipated to succeed FinFETs due to its ability to deliver enhanced device performance while maintaining reduced dimensions. The two main types of GAA devices are cylindrical (CGAA) and rectangular (RGAA). The CGAA device’s channel is distinguished by a cylindrical shape, whereas the RGAA device’s channel section has a rectangular geometry. This paper examines the rectangular GAA FET as the proposed device. GAA technology [12,13] demonstrates superior performance compared to TGFinFET technology regarding gate electric controllability and effectively mitigates various short-channel effects (SCEs). A GaN channel-based GAA nanobiosensor featuring uniform channel doping and high-k gate material at a channel length of 10 nm has been developed for the first time to compete with the IRDS 2025 [14]. This paper examines the performance of rectangular GAA as a biodetector.

Certain biomolecules possess a charge, while others remain neutral [15,16,17,18,19]. The characteristics that distinguish charged biomolecules from neutral biomolecules are charge density and dielectric constant. The electrical behavior of DM TGFinFETs may vary, suggesting the presence of specific biomolecules [20]. Several challenges, including the electrical properties of neutral biomolecules, have been taken into account. GaN, a III–V material, presents advantages over silicon as a channel material, as previously indicated by Saha et al. [21]. GaN was also observed to exhibit reduced sensitivity to performance degradation caused by temperature fluctuations compared to Si. This study looks at a simulation of GaN-based GAA FET and compares it to TGFinFET for use as a biosensor. The design of biosensors includes the detection and evaluation of enzymes, proteins, carbohydrates, and antibodies. This serves as the primary rationale for its application. The proposed GaN-based GAA biosensor, featuring a 10 nm channel length, is designed to detect biomolecules and analyze their impact on channel voltage and electrical characteristics. The electrical properties of biomolecules facilitate this task. Reducing scale presents challenges due to short channel effects (SCEs). Multi-gate devices such as FinFETs and GAA effectively address short-channel effects. GAA designs are simple to implement and effectively reduce the dimensions of biosensors while ensuring compatibility with CMOS technology.

Silicon (Si) is a commonly employed semiconductor material in electronics because of its advantages and natural oxide. However, its limited charge transport and maximal velocity hinder its use for modern electronic applications [22,23,24,25]. The increased power density of semiconductor circuits has led to higher operational temperatures. Researchers are exploring novel materials such as SiGe, SiC, and group III-V semiconductors to surmount these limitations. Gallium Nitride (GaN) and its associated alloys possess unique characteristics that suggest their viability for commercial applications, particularly in optoelectronic and high-power devices [26,27,28,29,30]. This proposed study utilizes GaN as the channel material.

This research has made major contributions, including the suggestion of a novel biosensor. This biosensor can manage leakage current better and have better sensitivity and linearity than other devices in the literature. The suggested device exhibits less performance decline when subjected to temperature variations than existing devices in use, according to a full examination.

## 2. Device Architecture

The purpose of this investigation is to fabricate a 14 nm tri-gate FinFET device on a GaN base that contains a buried oxide (BOX) layer, which is referred to as device A. A 14 nm GaN-based channel GAA FET is designated as device B, featuring Gaussian channel doping, while the same device with uniform channel doping is labeled as device C. Next, a GaN-based nanobiosensor with a 10 nm channel, called device D, has Gaussian channel doping, while another nanobiosensor with uniform channel doping is named device E.

**Figure 1:**
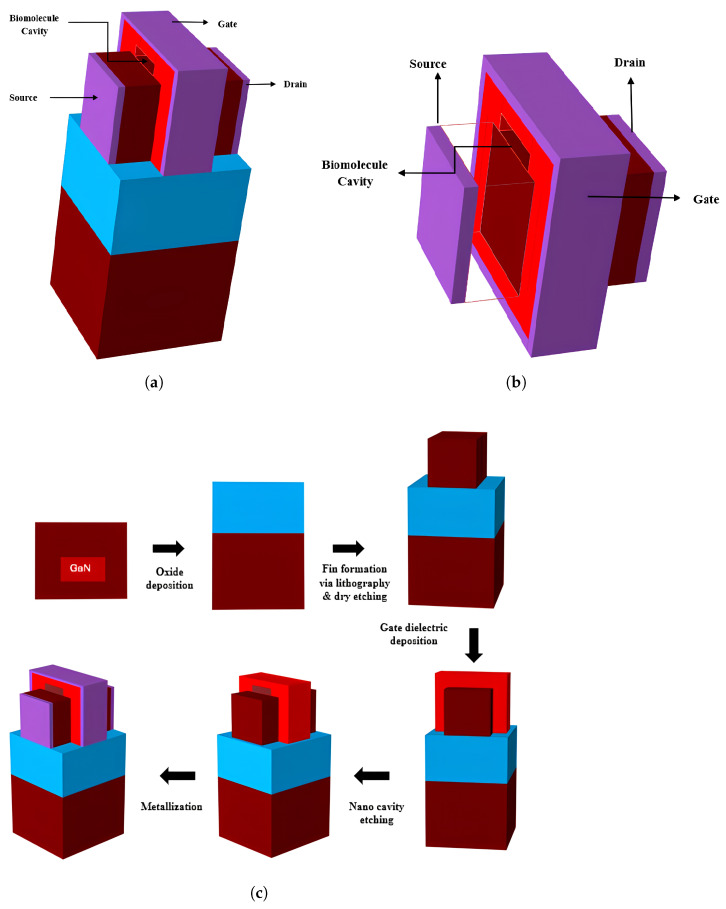
Proposed simulated device structure: (**a**) DM TGFinFET; (**b**) Nanobiosensor (10 nm gate length GAA FET); (**c**) Fabrication process diagram of proposed device.

Figure 1a illustrates a suggested biosensor utilising 3D TG-FinFET technology; Figure 1b represents the Gate All Around (GAA) architecture; and Figure 1c showcases nanobiosensor (GAA) technology. The fabrication procedure initiates the preparation of the substrate and the formation of the buried oxide (BOX) layer, subsequently proceeding to the deposition of a high-quality GaN layer utilizing either MOCVD or ALD techniques. Channel patterning and doping will be accomplished using ion implantation or in situ techniques. Subsequently, the nanocavity is precisely defined in the dielectric deposition section of the device as shown in Figure 1c through anisotropic dry etching methods carried out within the vacuum chamber by reactive ion etching (RIE) so as to avoid any molecular contamination. The deposition of HfO_2_ gate dielectric is then conducted using atomic layer deposition (ALD). Then, after the biomolecule is introduced into the nanocavity via controlled microfluidic delivery and is immobilized through surface functionalization with specific linker molecules that facilitate selective binding to target species while reducing non-specific adsorption using passivation layers of the nanocavity created within the vacuum chamber itself. Subsequently, the gate electrodes are formed and metallized and the Nanobiosensor device is developed. The device surface will then be subjected to passivation and the application of functionalization layers which are specifically designed for biomolecule detection. To transform all the above-mentioned devices into biosensors, we create a 4 nm cavity beneath the gate area. Table 1 presents the specs of the suggested devices. The primary difference between the traditional FinFET, GAA, and the proposed devices is in the integration of a cavity structure designed for biomolecule detection.

The Silvaco device simulator is employed for simulation applications. A physical model is crucial for the simulation of devices. The devices developed here employ both uniform doping as well as Gaussian channel doping. The Silvaco TCAD device simulator is utilized for the design and development of the devices. The carrier transport model is incorporated through the drift-diffusion model. In surface functionalization, molecular binding and charge screening effects are typically incorporated via interface charge models (INTERFACE) or interface trap models (TRAP), which are considered in the device development. The Local Material Model (LMM) is employed to analyze the impact of transverse field, doping, and temperature on mobility. The SRH (Shockley–Read–Hall) and Auger models are employed for carrier recombination analysis of the devices. The Fermi–Dirac approximation has been analyzed using the carrier concentration model while Boltzmann statistics are advisable for the carrier transport model and finally the automated Newton–Richardson method facilitates numerical convergence, which are all included in the simulation and thereby account for the biomolecule detection and further sensitivity analysis of the incorporated biomolecules in the devices. Meshing, material filling, and doping are the three stages involved in designing a GAA FET (device E) with a physical gate length of 10 nm. Depending on the material to be filled in the specific sections of the created meshes, the structure is divided into different areas. The device uses polysilicon as the gate contact, GaN as the dopant, and HfO_2_ as the gate dielectric.

The proposed GaN-based biosensors identify biomolecules and examine how they affect electrical properties and channel voltage. After adding several biomolecules close to the cavity structure, this study notes and explains the variation in electrical properties. Current can alter the implanted biomolecules’ ion charge and dielectric characteristics. FETs may be smaller now, which makes detection simpler. Biomolecules may settle more easily in the proposed device thanks to nanocavity gaps. The type of biomolecule present may be indicated by changes in the suggested device’s electrical activity.

Channel engineering, which is used in a variety of FETs, provides a clear explanation of the features of a FET by reducing the channel electric field significantly in comparison to the vertical electric field using 1D Poisson’s equation. Because it impairs the device’s performance depending on the current passing through it, the leakage current produced in the off-state condition is undesirable. For uniform $V_{GS}$, the subthreshold voltage results in conversion from the depleted channel to the inverted channel. The subthreshold current is the drain current obtained at this point, which persists for $V_{GS}$ << $V_{TH}$. Thus, the following specifies the subthreshold drain current:

(1) $$I_{SDC}\;=\;Z{\left(KT/q\right)}^{2}e^{q(V_{GS}-V_{TH})/\eta KT}\left(1-e^{-qV_{DS}/KT}\right)$$

where $Z\;=\;\mu_{effec}.C_{OX}.\frac{W_{CH}}{L_{CH}}.\left(n-1\right)$

When the $V_{DS}$>(KT/q). Consequently, the following is obtained:

(2) $$I_{SDC}\;=\;Z{\left(KT/q\right)}^{2}e^{q(V_{GS}-V_{TH})/\eta KT}$$

The subthreshold current is independent of $V_{GS}$ and $V_{DS}$, and steadily decreases with decreasing $V_{GS}$. When $V_{GS}$ = 0 and $V_{DS}$ = $V_{DD}$, $I_{OFF}$ is the $I_{D}$, is defined as $I_{SDC}$. The $I_{OFF}$ is determined based on characteristics including channel dimensions, source/drain junction depth, gate oxide width, and doping concentration. The mobility of charge carriers may be determined using the following generic formulae [31]:

(3) $$\mu_{effec,n}\;=\;\frac{638}{1+\frac{E_{effec}}{7\times 10^{5}}}$$

(4) $$\mu_{effec,p}\;=\;\frac{240}{1+\frac{E_{effec}}{2.7\times 10^{5}}}$$

where $E_{effec}$ is the effective channel electric field of the proposed device. In case when $V_{GS}$ > $V_{TH}$, $I_{SDC}$ is given by

(5) $$I_{SDC}\;=\;Z\left[\left(V_{GS}-V_{TH}\right)V_{DS}-\left(\eta V_{DS}^{2}\right)/2\right]$$

In a linear system the drain current ($I_{D}$) can be stated as

(6) $$I_{SDC}\;=\;Z\left[\left(V_{GS}-V_{TH}\right)V_{DS}\right]$$

**Figure 2:**
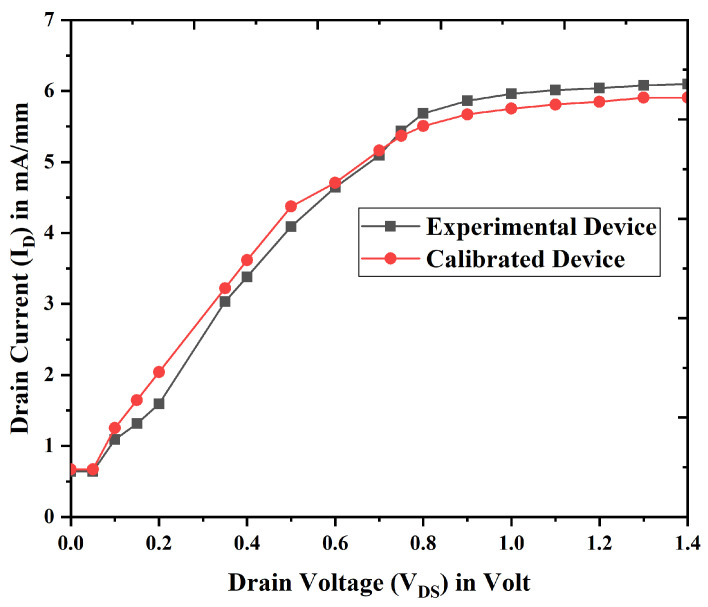
Calibration model against experimental data [32] for $V_{GS}$ = 1.5 Volt.

The computed drain current for device E (Nanobiosensor) is 5.02 mA/µm, while the simulated value is 5.07 mA/µm. The GaN FinFET (gate length 20 nm, cavity 8 nm) experimentally fabricated by Hisamoto et al. [32] is used for calibration. Considering this, a similar device of 20 nm gate length is developed using Silvaco TCAD simulation. The $I_{D}$ –$V_{DS}$ plot is generated at a $V_{GS}$ of 1.5 V, which almost matches closely with the experimental data for the existing device and is presented in Figure 2. Thus, experimentally produced 20 nm gate length GaN FinFET is used to calibrate and validate the system for further device design and analysis, suggesting that the simulation is accurate and efficient and is applicable for experimental fabrication of the proposed device. Next, qualitative and quantitative studies are conducted on the recently designed 14 nm DM TGFinFET devices, and estimations for short channel effects such SS and DIBL are carried out.

## 3. Results and Discussion

There are both neutral and charged forms of biomolecules. Utilizing the dielectric constant is a method of investigating neutral biomolecules. Furthermore, the recommended device model regards blood as a biomolecule. At 78, the dielectric constant of blood is the highest available. When no biomolecules are present, the air-filled cavity has a dielectric constant of 1. The electrical properties of the device were examined to identify biomolecules. These characteristics included the ratio of $I_{on}$/$I_{off}$, Vth, SS, and DIBL. Research has been conducted to investigate the impact that air in the cavity has on a wide range of biomolecules. The sensitivity parameter is important and heavily weighted in the scientific community when it comes to biomolecule detection.

### 3.1. Electrical Characteristics and Analysis of Nanobiosensor

The effects of channel doping, channel length, and high-K material induction on a range of physical characteristics of recently developed 10 nm nanobiosensors are examined in this work. A number of critical characteristics are investigated by taking into consideration the various channel doping, as well as the reduction in channel length of the devices that have been produced. These parameters include drain current versus gate voltage $I_{D}$-$V_{GS}$, $I_{D}$-$V_{DS}$, on-current, off-current, and the switching ratio.

**Figure 3:**
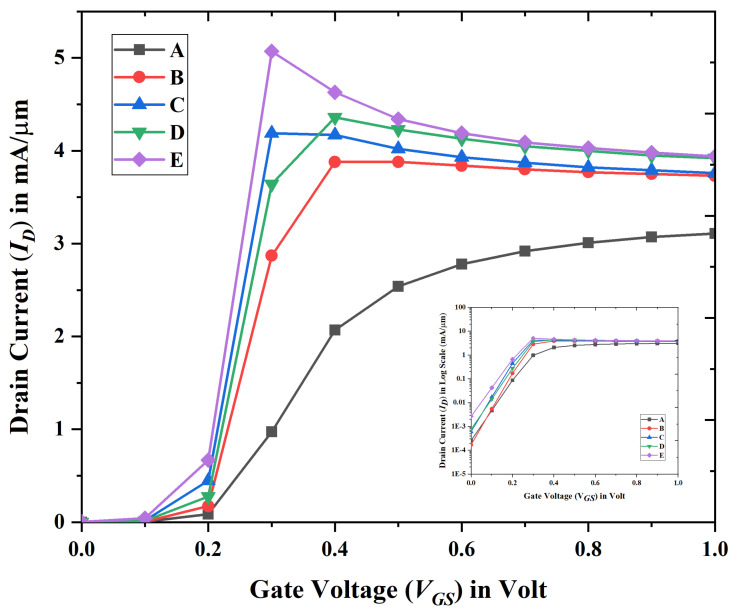
Linear scale transfer characteristics for device A, B, C, D, and E at $V_{DS}$ = 0.1 volt when T = 280 K. (Inset) drain current in log scale.

Figure 3 illustrates the fluctuation of the transfer characteristics curve for the various devices that are presented in Table 1 when the voltage from the drain to the source is kept constant at 0.1 volts.

The graph makes it clear that the performance of the Nanobiosensor with uniform channel doping is superior to that of the TGFinFET and the GAA FET. It has been observed that the drain current is in the range of µA for the temperature variation which is same in order. Therefore, there has not been a discernible change in the drain current that has been detected in relation to the temperature shift. According to the findings of the investigation that has been carried out, the device has been depicted to be independent of change in temperatures.

**Figure 4:**
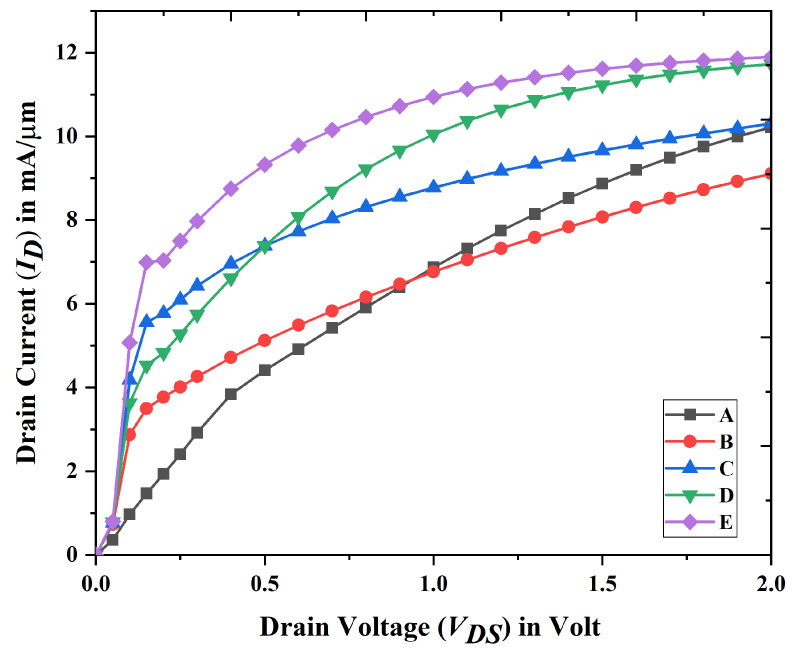
Drain current variation in drain characteristics curve for device A, B, C, D, and E at $V_{GS}$ = 0.5 volt when T = 280 K. (Inset) drain current in log scale.

It can be shown in Figure 4 that the suggested device E, which is a Gate All Around Nanobiosensor FET, has attained its maximum drain current in comparison to other devices.

**Figure 5:**
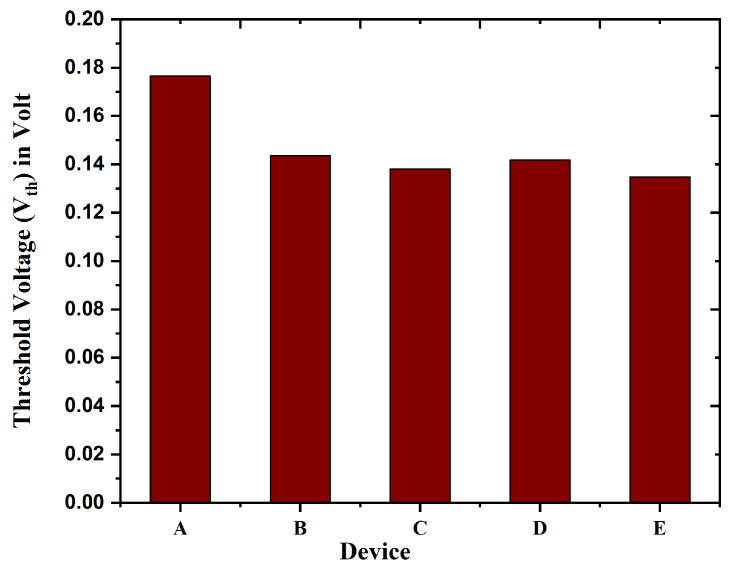
Comparison of threshold voltage variation ($V_{th}$) for device A to E.

Though TGFinFETs and nanoscale biosensors (GAA) both display threshold voltage changes, the reasons and implications of these variations are distinct from one another. There are a few different reasons that might cause variances in TGFinFET. These include gate-induced effects, random doping fluctuations, and process variations. Alterations in threshold voltage are frequently a significant sensing mechanism in biosensors. These changes are a reflection of the interaction that occurs between the device and the biomolecules that are being targeted. A representation of the minimal threshold voltage ($V_{th}$) that has been acquired for the Nanobiosensor (device E) can be observed by looking at Figure 5. Furthermore, the threshold voltage can be influenced by the work function ($\phi_{F}$) of the gate material and the gate-to-channel interface. The $\phi_{F}$ of the gate in this study is measured at 4.6 eV.

**Figure 6:**
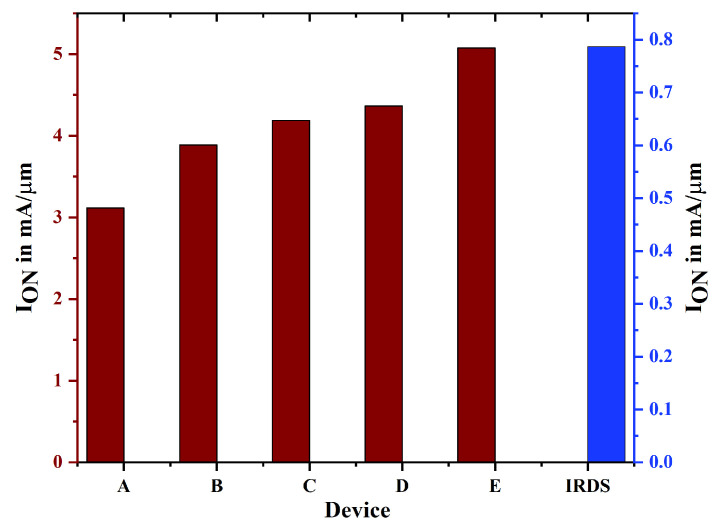
$I_{ON}$ of the device E, D, C, and B are assessed with the proposed data of IRDS 2025 and the existing device.

**Figure 7:**
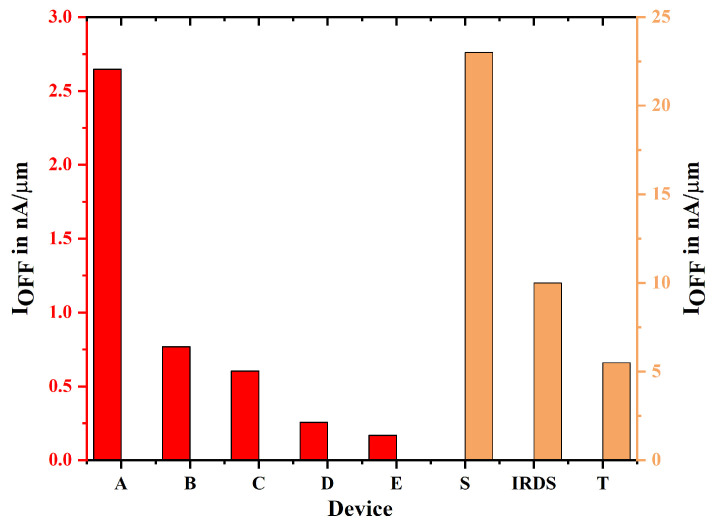
The leakage current for device A to E is assessed with the existing device S, device T, and IRDS 2025.

In TGFinFET and GAA (Gate-All-Around) transistors, the “on” current is defined as the current that flows through the channel when the gate voltage is elevated, thereby activating the transistor. GAA devices, particularly those featuring nanostructures, offer advantages over TGFinFETs in terms of regulating on-current and minimizing leakage (Figure 6 and Figure 7). In GAA devices, the on-current can be adjusted by altering the width of the GAA structure, unlike TGFinFETs, where it is constrained by the number of fins. This study illustrates that the GAA nanostructure with uniform doping thereby displays a higher on-current compared to other devices. TGFinFETs feature a gate that encases the channel on three sides, whereas GAA FETs encompass the channel on all four sides, which leads to enhanced gate control. The better gate control of GAA FETs means less unwanted current flows when the transistor is off, as it keeps the channel well separated from the source and drain. Even though TGFinFETs are better than regular planar transistors, they still have more off-current than GAA FETs because leakage happens through the channel, especially at the edges of the fins. A reduction in off-current in GAA FETs leads to lower power consumption, especially in low-power applications where static leakage power is critical.

**Figure 8:**
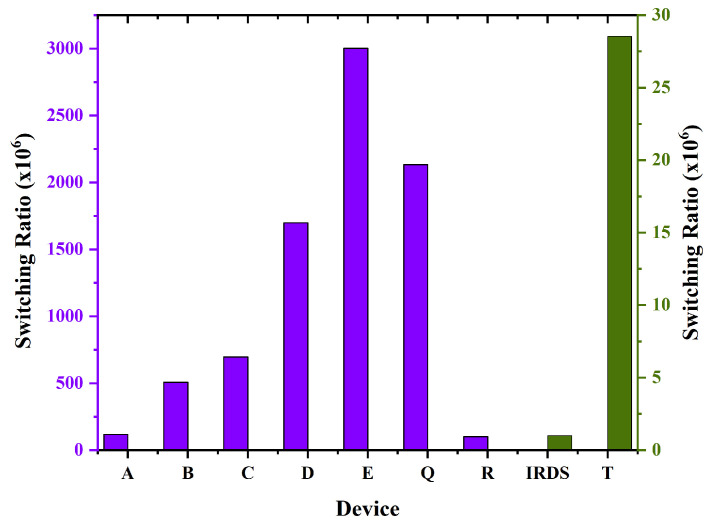
Comparison of switching ratio of device A to E with the existing device R, device T, and IRDS 2025.

GAA FETs, particularly Nano FETs with uniform channel doping, are anticipated to exhibit a higher switching ratio compared to FinFETs, attributed to their enhanced electrostatic control and capability to mitigate short-channel effects. The complete gate surround in GAAFETs facilitates enhanced control over the channel region, in contrast to the three-sided gate configuration found in FinFETs. FinFETs represent a notable improvement compared to planar transistors; however, GAA FETs are expected to offer enhanced efficiency and scalability for upcoming generations of chip technology. In Figure 8, Gate-all-around (GAA) Nanobiosensor field-effect transistors (FETs) demonstrate elevated switching ratios, indicating a substantial difference between the on current ($I_{on}$) and the off current ($I_{off}$). The devices, especially those incorporating nano structures, are capable of attaining switching ratios on the order of $10^{6}$. The superior gate control over the channel facilitates a significantly sharper current transition between the on and off states.

**Figure 9:**
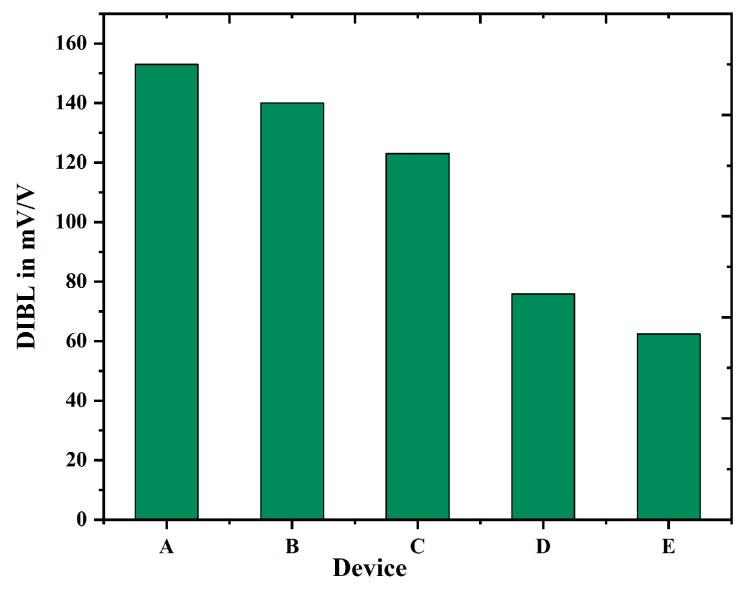
DIBL comparison of device A to E.

**Figure 10:**
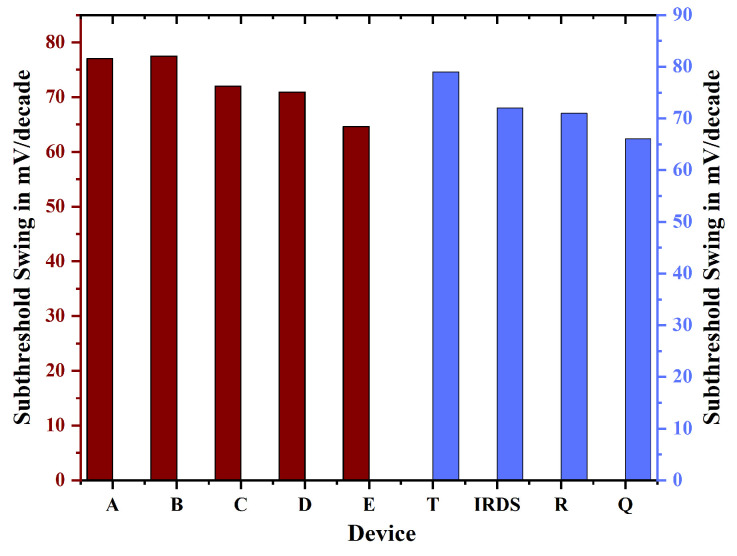
The subthreshold swing for devices A through E is assessed with the T, R, Q, and IRDS 2025.

Gate-all-around (GAA) FETs typically demonstrate reduced Drain-Induced Barrier Lowering (DIBL) in comparison to TGFinFETs, attributable to their enhanced gate control over the channel. This is due to the configuration of GAA FETs, which encase the channel on all sides, in contrast to FinFETs that only encase it on three sides. The improved gate control in GAA FETs results in more effective suppression of short-channel effects such as DIBL. DIBL denotes the phenomenon where the $V_{TH}$ of a transistor decreases as the $V_{DS}$ is elevated. The observed effect results from the reduction in the drain-induced potential barrier, facilitating an increased current flow through the channel, even when the $V_{GS}$ is below the threshold level. The TGFinFET configuration features a fin channel that is enveloped by the gate on three sides. Although this configuration enhances gate control compared to a planar MOSFET, it does not achieve the same degree of control as a GAA FET. Figure 9 depicts that Nanobiosensor with uniform channel doping (device E) provides better control over other devices because reduced DIBL enhances transistor performance and decreases power dissipation. Through the mitigation of short-channel effects such as DIBL, GAA FETs demonstrate enhanced reliability and extended device longevity. The diminished effect of DIBL facilitates improved scaling of transistors while maintaining performance integrity. Nanobiosensor (GAA) FETs provide a notable advantage in subthreshold swing (SS) compared to TGFinFETs, attributed to their enhanced gate control. This results in decreased leakage current and improved on/off switching performance. Subthreshold swing (SS) quantifies the variation in $I_{DS}$ in relation to $V_{GS}$ when the transistor is in its off-state. A steeper SS signifies a more rapid transition from the off state to the on state, accompanied by a reduction in leakage current, as illustrated in Figure 10. Table 2 provides a detailed comparison between the recently introduced device E alongside the existing literature.

### 3.2. Sensitivity and Limit of Detection (LOD) Analysis

**Figure 11:**
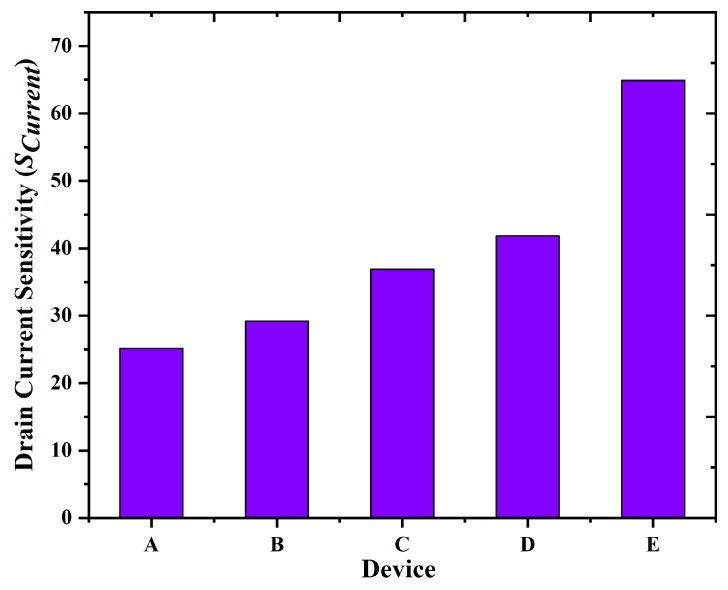
Comparison of sensitivity profile for device A to E.

The sensitivity parameter (S) is a primary metric for assessing a biosensor’s ability to detect biomolecules. Equation (7) outlines the methodology by which the proposed GaN-based nanobiosensor assesses sensitivity through the comparison of drain current ($I_{D}$) prior to and subsequent to biomolecule conjugation. In the detection of blood as the target biomolecule, device E (GAA nanobiosensor with uniform doping), as shown in Figure 11, exhibits higher current sensitivity among all the devices analyzed reaching approximately 65 at $V_{GS}$ = 0.5 V. This high sensitivity enables the identification of biomolecules at very low concentrations, which is crucial for applications such as in food safety testing, environmental monitoring, and early disease diagnosis.

(7) $$S_{current}\;=\;\frac{I_{D}^{Bio}-I_{D}}{I_{D}}$$

where $I_{D}^{Bio}$ indicates drain current after biomolecule conjugation, and $I_{D}$ is the drain current before biomolecule conjugation.

**Figure 12:**
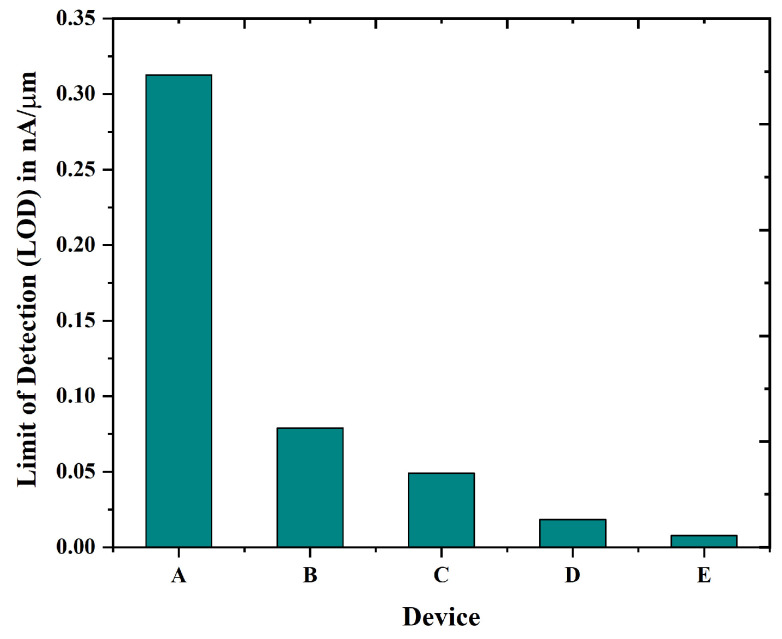
Comparison of limit of detection for device A to E.

The limit of detection (LOD) serves as a vital performance metric for biosensors, indicating the minimum concentration of an analyte that can be reliably identified by the sensor, usually differentiated from the background signal or noise. In biosensing applications, a lower limit of detection signifies a more sensitive device that can identify trace amounts of biomolecules like proteins, DNA, toxins, or viruses. This sensitivity is particularly crucial in fields such as medical diagnostics, environmental monitoring, and food safety. The limit of detection (LOD) is typically calculated using the standard deviation of the baseline signal (air-filled cavity) and the sensor’s sensitivity, according to the formula:

(8) $$LOD\;=\;\frac{3\times \sigma }{S}$$

where σ represents the standard deviation of the base line signal (air-filled cavity) and S denotes the sensitivity of the device. In biosensors that utilize field-effect transistors (FETs), the limit of detection (LOD) can be determined by observing alterations in drain current or threshold voltage as analyte concentrations fluctuate. Attaining a low limit of detection frequently requires the enhancement of the sensor’s surface functionalization, improvement in transducer efficiency, and implementation of noise reduction strategies. Ultimately, the limit of detection serves as a benchmark for comparing biosensor performance and ensuring reliability in detecting low-abundance targets. In Figure 12, the graphical representation shows that the LOD varies with the different devices. As seen, the nanobiosensor (device E) indicates better sensitivity and detectibility.

Simulated findings show that, when blood is present in the nano-cavity across all devices, device E, which is a nanobiosensor, has a substantially greater $S_{current}$ than the others, reaching nearly 65 at $V_{GS}$ = 0.5 volts, as illustrated in Figure 11. The sensitivity of the proposed device has been evaluated in comparison to all other devices, revealing that the proposed device exhibits significantly greater sensitivity for biomolecules. For the detection of tiny levels of analytes, which are important in many applications such as food safety, environmental monitoring, and medical diagnostics, high sensitivity is essential. In this work, the proposed GAA Nanobiosensor has the highest sensitivity among the all other devices.

**Figure 13:**
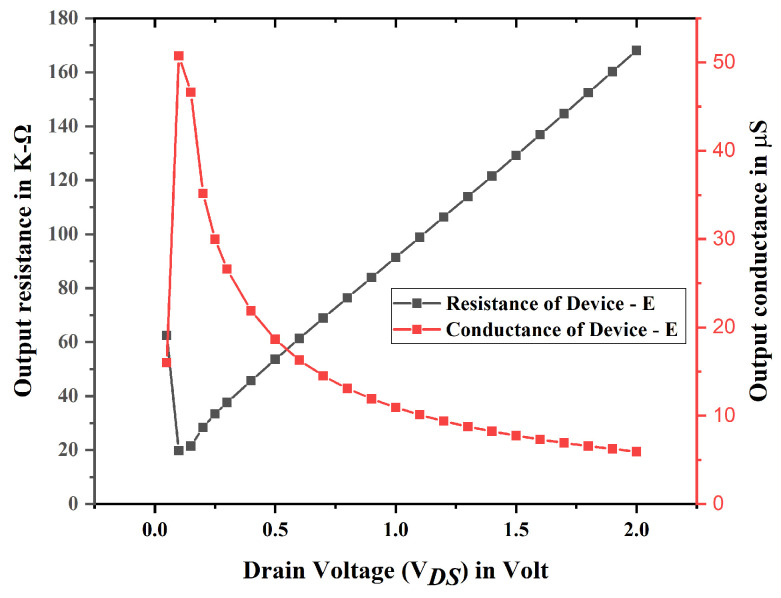
Resistance and conductance profile for device E.

Figure 13 illustrates the variations in the output characteristics of the proposed model in relation to drain voltage, with a constant gate to source voltage set at 0.1 volt. The drain conductance shows a linear increase at low drain voltage. For larger drain voltages, the system reaches a saturation point and maintains its minimum value. This illustrates the minimal effect of drain voltage on the tunneling process.

TGFinFETs and GAA FETs with uniform channel doping vary in their channel regulation and resistance characteristics. TGFinFETs, characterized by three-sided gate control, exhibit increased source/drain resistance and heightened vulnerability to parasitic effects. GAA FETs, with complete gate wrap-around, provide increased electrostatic control, resulting in reduced leakage currents and improved performance at smaller dimensions. GAA FETs are capable of superior drive current and diminished latency relative to FinFETs at lower dimensions. Figure 12 illustrates the variations in the output characteristics of the proposed model concerning drain voltage, with a constant $V_{GS}$ of 0.1 volt. At low drain voltage, the drain conductance demonstrates a linear increase. However, at elevated drain voltages, it has attained a saturation point and sustains its minimum value. This illustrates the little influence of drain voltage on the tunneling process.

### 3.3. Linearity Analysis

In practical biosensing systems, particularly for portable or wireless applications, the transducer is typically succeeded by analog amplification and potentially high-frequency readout circuitry. Poor linearity in the sensor or front-end electronics can be characterized by low VIP2, VIP3, IIP3, which result in distortion components that obscure the true sensor signal. This phenomenon adversely affects detection accuracy, diminishes the limit of detection (LOD), and increases false-positive rates. Hence, the analysis and an enriched performance of VIP2, VIP3, IIP3 guarantees that the biosensor device output can accurately reflect the true biomolecule interaction signal, despite the presence of significant interferences, substantial signal fluctuations, or environmental electromagnetic noise which are needed for betterment of the device.

**Figure 14:**
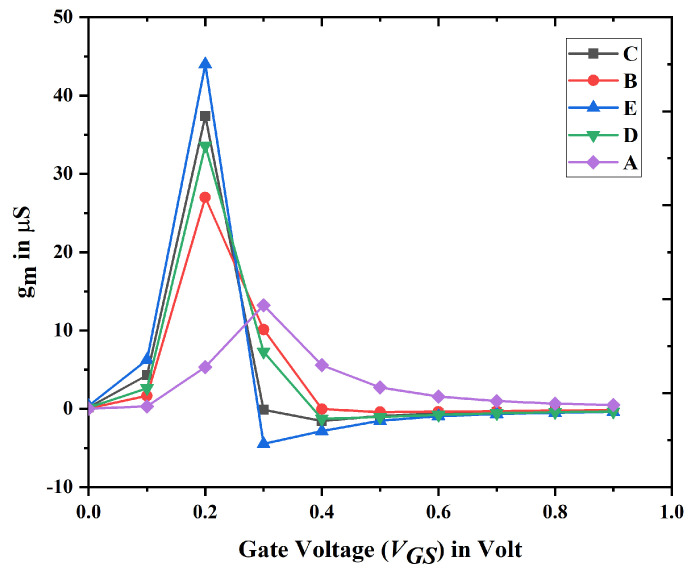
Transconductance ($g_{m1}$) for device A to E.

An optimal biosensor must have a high level of linearity and little distortion. The deterioration of device linearity may lead to a drop in SNR, hence diminishing the sensitivity of biosensors. These parameters have been evaluated through the calculation of transconductance. Figure 14 illustrates the transconductance of devices A to E. The linearity of Nanobiosensor (device E) has been assessed using $g_{m1}$, $g_{m2}$, $g_{m3}$, VIP2, VIP3, IIP3, and the P1dB. In the design of generic RFICs, minimizing third-order nonlinearity is vital for reducing intermodulation distortion. The VIP2, VIP3, IIP3, and P1dB must be optimized to attain superior linearity. General form of transconductance for any FET is given by Equation (9).

(9) $$g_{mn}\;=\;\frac{\partial^{n}I_{DS}}{\partial {V_{GS}}^{n}}$$

Since the higher-order transconductance controls the distortion restriction, $g_{m2}$ and $g_{m3}$ must have low amplitudes in order to achieve minimum distortion. At the Zero-Crossing point (ZCP), which is located on the $V_{GS}$ axis, third-order transconductance ($g_{m3}$) is null. The ZCP should be close to the $V_{TH}$ as it determines the DC bias point. Robust linearity at low $V_{GS}$ levels is ensured by the zero crossover point’s smallest magnitude. Improved linearity at low voltage bias is advantageous for a portable radio frequency application.

**Figure 15:**
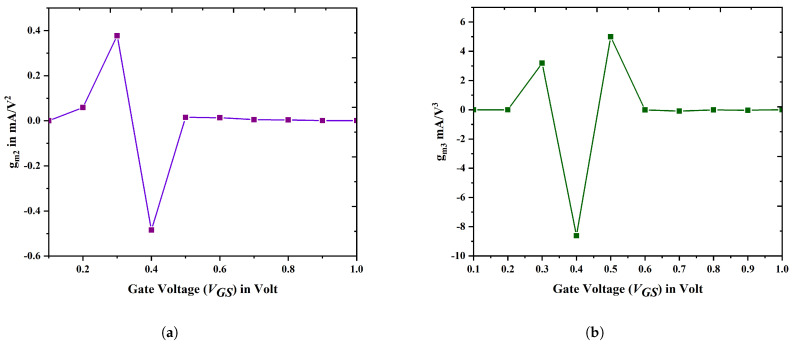
Transconductance derivatives of device E: (**a**) Second-order transconductance; (**b**) Third-order transconductance for different $V_{GS}$.

Figure 15a,b illustrate the higher orders of transconductance, specifically the second and third, in relation to the gate-to-source voltage. The proposed device exhibits a reduction in the higher order transconductance value. The Zero Crossover Point (ZCP) represents the specific value of $V_{GS}$ at which both $g_{m2}$ and $g_{m3}$ reach zero. This point is critical for establishing the DC bias point necessary for device operation. The Zero Crossover Point is minimized for the proposed DM TGFinFET, resulting in decreased power consumption across a range of circuit applications.

**Figure 16:**
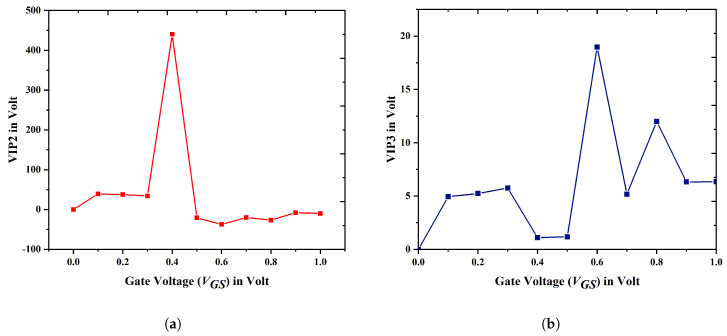
Variation in (**a**) voltage intercept point 2 (VIP2); (**b**) voltage intercept point 3 (VIP3) for device E at different $V_{GS}$.

Figure 16a,b depict the variations in the voltage intercept points for the second and third orders across different levels of the gate-to-source voltage. The VIP2 (450 V) and VIP3 (43 V) values demonstrate superiority for the proposed device in comparison to the other two devices [37,38], signifying improved linearity and diminished distortion performance. The comparison of various devices regarding linearity is presented in Table 3. The increased concentrations of VIP2 and VIP3 have a direct impact on carrier velocity, thereby affecting the transconductance of the device.

**Figure 17:**
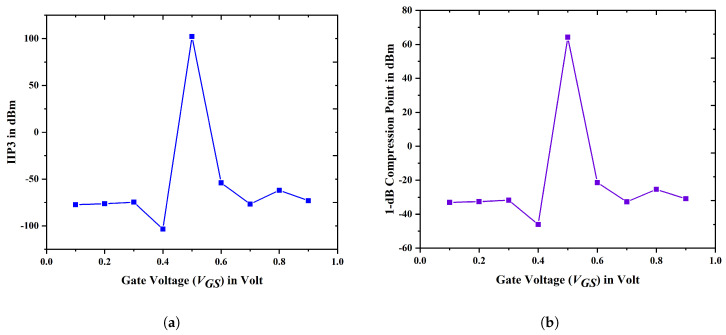
Variance in (**a**) IIP3; (**b**) P1dB with different $V_{GS}$ values.

Figure 17a demonstrates the enhancement of IIP3 in device E, primarily due to the increased transconductance and decreased $g_{m3}$. This subsequently improves the transit efficiency of the carriers, thereby enhancing the overall gate controllability within the channel region. Figure 17b illustrates the changes in the 1 dB compression point of the proposed device E across different gate voltages. The 1 dB compression point represents the specific threshold where distortion initiates and compression starts to occur. The 1 dB compression point should exhibit an increased value to improve linearity. It is advantageous that the suggested device has a 1 dB compression point value which is marginally greater than the other devices, as shown in Figure 17b. A major contributing aspect to this result is the proposed device’s higher gm value. Table 2 provides an analysis of the linearity of the novel device E with the existing literature.

The developed device operates at a low threshold voltage of approximately 0.18 V compared to a partial recessed gate HEMT biosensor (Wang et al.) [39] with a threshold voltage of approximately 2 V, which makes the proposed device developed here more suitable for low-power applications and the electrical parameters hence stand to be superior to those of the referenced devices. Therefore, it offers better performance for the intended application being compatible with the CMOS-based semiconductor industry.

## 4. Conclusions

The effort to expand the use of biosensing has led to the creation of a GaN-based GAA nanobiosensor FET. The investigation of electrical activity has led to the discovery of several biomolecules that are entirely distinct. Several methods are used to study these biomolecules including ON-OFF current, switching ratio, sensitivity, subthreshold slope, and DIBL, among others. The results obtained clearly indicate that using GaN as a channel material led to a significant enhancement in the electrical performance of the GAA nanobiosensor, particularly with uniform channel doping. This study shows that device E lowers the OFF current and threshold voltage by about ∼60% and ∼65%, respectively, compared to device Q, which matches the limits recommended by IRDS 25. Additionally, the new device shows about a ∼50% decrease in subthreshold swing, with DIBL measured at 62.4 mV/V, which means it handles short-channel effects better. The sensitivity of the biosensor improves with a reduction in the gate length of the GAA FET compared to the biosensor currently in use. The findings of this study demonstrate that GaN-based DM TG-FinFET can effectively serve as a biosensor, proving advantageous for various sensing applications. This is because it can detect different biomolecules very well while using little power, which helps to identify a wide range of related diseases. Additionally, the lower Zero Crossover Point (ZCP) and higher peak values of VIP2, VIP3, and IIP3 show that there is less distortion and better linearity. The proposed device shows major improvements in features like VIP2 (about ∼120%), VIP3 (about ∼79%), and IIP3 (about ∼200%) compared to GaN-based SOI FinFET, as shown in Table 3. The GAA nanobiosensor showcases its advanced capabilities as a biosensor, positioning it as the leading choice for future applications.

## Figures and Tables

**Table 1:** Device specifications with dimensions.

Device	Specification of Device	Channel Doping	Channel Length	Source and Drain Length
A	TGFinFET	Gaussian	14 nm	10 nm
B	GAAFET	Gaussian	14 nm	10 nm
C	GAAFET	Uniform ($10^{15}$ atoms/CC)	14 nm	10 nm
D	Nano Biosensor (GAA)	Gaussian	10 nm	8 nm
E	Nano Biosensor (GAA)	Uniform ($10^{15}$ atoms/CC)	10 nm	8 nm

**Table 2:** Comparative analysis of electrical parameters between the proposed device and other devices.

Device Abbreviation	$I_{\mathrm{ON}}$ (mA/μm)	$I_{\mathrm{OFF}}$ (nA/μm)	Switching Ratio (nA/μm)	Subthreshold Voltage	DIBL
A	3.113	2.647	1.176	77	153
B	3.884	0.767	5.06	77.5	140
C	4.186	0.602	6.953	72	123
D	4.364	0.257	16.98	70.9	75.9
E	5.072	0.169	30.01	64.6	62.4
Q [33]	2.5	0.11	21.34	66	Not Given
R [34]	1.2	1.2	1	71	Not Given
S [13]	0.037	23	0.0016	0.07	Not Given
T [35]	1.57	5.5	28.54	79	Not Given
IRDS 2025 [14]	0.787	10	0.787	72	Not Given
P [36]	4.46	4.86	9.18	65.03	Not Given

**Table 3:** Analysis of several linearity results for distinct devices.

Device	$g_{m2}$ (mA/V^2^)	$g_{m3}$ (mA/V^3^ )	VIP2 (V)	VIP3 (V)	IIP3 (dBm)
Conventional FinFET [37]	1	7.8	50	3	Not given
GaN SOI FinFET [38]	0.3	2	100	24	24
E	0.39	5	450	20	98

## Data Availability

The datasets used and analyzed during the current study are available from the corresponding author upon reasonable request.
